# Radiation Dosimetry of a Novel Adenosine A_2A_ Receptor Radioligand [^11^C]Preladenant Based on PET/CT Imaging and Ex Vivo Biodistribution in Rats

**DOI:** 10.1007/s11307-016-0992-3

**Published:** 2016-08-18

**Authors:** Xiaoyun Zhou, Philip H. Elsinga, Shivashankar Khanapur, Rudi A. J. O. Dierckx, Erik F. J. de Vries, Johan R. de Jong

**Affiliations:** Department of Nuclear Medicine and Molecular Imaging, University of Groningen, University Medical Center Groningen, Groningen, The Netherlands

**Keywords:** Radiation dosimetry, [^11^C]preladenant, Small-animal PET/CT, Adenosine A_2A_ receptor, Rat

## Abstract

**Purpose:**

[^11^C]Preladenant was developed as a novel adenosine A_2A_ receptor PET radioligand. The aim of this study was to determine the radiation dosimetry of [^11^C]preladenant and to investigate whether dosimetry estimation based on organ harvesting can be replaced by positron emission tomography (PET)/x-ray computed tomography (CT) imaging in rats.

**Procedures:**

Male Wistar rats (*n* = 35) were *i.v.* injected with [^11^C]preladenant. The tracer biodistribution was determined by organ harvesting at 1, 5, 15, 30, 60, and 90 min post injection. Hollow organs including the stomach, intestines, and urinary bladder were harvested with contents. In 10 rats, a 90-min dynamic PET/CT scan of the torso was acquired. Twenty volumes of interest (VOIs) were manually drawn on the PET image using the CT image of the same animal as anatomical reference. The dynamic time-activity curves were used to calculate organ residence times (RTs). Human radiation dosimetry estimates, derived from rat data, were calculated with OLINDA/EXM 1.1.

**Results:**

PET-imaging and organ-harvesting estimated comparable organ RTs, with differences of 6–27 %, except for the lungs, pancreas, and urinary bladder, with differences of 48, 53, and 60, respectively. The critical organ was the small intestine with a dose of 25 μSv/MBq. The effective doses (EDs) calculated from imaging-based and organ-harvesting-derived data were 5.5 and 5.6 μSv/MBq, respectively, using the International Commission on Radiological Protection 60 tissue weighting factors.

**Conclusions:**

The ED of [^11^C]preladenant (2 mSv for a 370-MBq injected dose) is comparable with other C-11-labeled PET tracers. Estimation of the radiation dosimetry of [^11^C]preladenant by PET/CT imaging in rats is feasible and gives comparable results to organ harvesting, provided that small VOIs are used and the content of hollow organs is taken into account. Dosimetry by PET imaging can strongly reduce the number of laboratory animals required.

**Electronic supplementary material:**

The online version of this article (doi:10.1007/s11307-016-0992-3) contains supplementary material, which is available to authorized users.

## Introduction

The adenosine A_2A_ receptor (A_2A_R) has been studied as a potential therapeutic target in peripheral inflammatory diseases [1] and in brain disorders, such as depression [2], drug addiction [3], Alzheimer’s disease [4], and Parkinson’s disease [5]. We have recently synthesized [^11^C]preladenant, a novel positron emission tomography (PET) radioligand for the imaging of A_2A_R in the central nervous system [6]. The tracer displayed excellent target-to-non-target ratios as well as favourable pharmacokinetic profiles, which warrants its translation to studies in human subjects.

Before performing a clinical study, a radiation dosimetry estimation is necessary to determine the dose limit of a new radiopharmaceutical [7]. Experimental animals have been used to estimate the radiation burden of a new tracer in humans. Preclinical radiation dosimetry measurements are often performed in non-human primates with PET imaging, and in rodents with organ harvesting at several time points post injection [8–10]. Due to the influence of the animal rights movement, studies on non-human primates are subject of discussion, while conventional ex vivo organ dissection in rodents requires a large number of animals (i.e., 24 animals for a typical ex vivo biodistribution study) to obtain the dynamic biodistribution data of radiopharmaceuticals required for dosimetry calculations. With the improvement of spatial resolution of the PET camera, small-animal PET imaging emerges as a promising alternative to study tracer distribution in vivo [11–14]. Dynamic PET-imaging enables studying tracer biodistribution over time using a single animal with much higher time resolution compared with organ-harvesting. The first dosimetry study with PET imaging in rodents was reported by Palm et al. [15], using Y-86 Trastuzumab to estimate the absorbed doses of [^90^Y]Trastuzumab in tumor and several organs in tumor-bearing nude mice. Absorbed doses in mice were calculated following the Medical Internal Radiation Dose (MIRD) method, using the murine-specific S factors. However, dynamic PET imaging data from previous studies showed significant deviation from organ harvesting in multiple organs, probably due to the small organ sizes comparative to the low spatial resolution of the PET camera, resulting in a severe partial volume effect (PVE)/spillover effect on imaging data. Moreover, due to loss of contents of hollow organs during the organ dissection, large discrepancies were found in the measurements of hollow organ activities between the two methods, as PET-imaging gives a measure of organ walls and contents while the organ-harvesting usually ignores the contribution of the organ contents.

In the present study, we determined the maximum injected dose of [^11^C]preladenant for humans based on whole-body PET imaging and ex vivo biodistribution in rats. In order to minimize the PVE/spillover effects, high-resolution small-animal x-ray computed tomography (CT) was applied to aid with organ delineation. Furthermore, spherical volumes of interest (VOIs) were drawn at the center of the organs to further reduce PVE/spillover effect. Finally, all hollow organs were dissected with contents; therefore, the negative bias on activity estimates in hollow organs due to loss of contents was avoided.

## Materials and Methods

### Radiochemistry

[^11^C]Preladenant was synthesized as described by Zhou et al. [6]. The radiochemical purity of [^11^C]preladenant was always greater than 98 %. The specific activity of the product was 122 ± 28 GBq/μmol (*n* = 11). [^11^C]Preladenant was formulated in phosphate buffered saline (pH = 7.4) to give the final product ready for injection.

### Animals

Adult male Wistar rats (Hsd/Cpb:WU, Harlan, the Netherlands, 291 ± 18 g, *n* = 35) were housed in groups at a 12 h light/12 h dark circle. The animals were fed with standard laboratory chow (RMH-B, The Netherlands) and water ad libitum. After arrival, the rats were allowed to acclimatized for at least 7 days. The research protocols were approved by the Institutional Animal Care and Use Committee of the University of Groningen (DEC 6689H).

### Ex vivo Biodistribution

[^11^C]Preladenant (18 ± 10 MBq (0.15 ± 0.09 nmol) for organ-harvesting only, 54 ± 20 MBq (0.48 ± 0.12 nmol) for organ harvesting combined with 90 min PET-imaging) was *i.v.* administered to rats (*n* = 5 per time point for time points 1–60 min, *n* = 10 for time point 90 min) via tail vein under isoflurane anesthesia (isoflurane in oxygen at a flow rate of 0.8 l/min, 5 % isoflurane for induction, 1.5–2.5 % isoflurane for maintenance). At 1, 5, 15, 30, 60, and 90 min post injection, animals were sacrificed by extirpation of the heart. Major tissues (brain, heart, lungs, liver, stomach, spleen, pancreas, kidney, small intestine, large intestine, testes, and bladder) were harvested and weighted, and the activity was measured using a calibrated well counter (2480 Wizard^2^, PerkinElmer, Waltham, MA, USA). The hollow organs (i.e., heart, intestines, stomach, and urinary bladder) were dissected with contents. Activity in the stomach and intestines was corrected for geometry effects due to the large volumes of these organs (with contents), using the correction factors presented in Supplementary Fig. 1. The data was expressed as Becquerel per gram tissue (Bq/g) and then normalized to body weight and injected dose to obtain standardized uptake values (SUVs) or converted to percent inject dose per tissue (%ID). The data were corrected for radioactive decay to the time of injection.

### PET/CT Acquisition

During the PET/CT scan, animals were anesthetized with isoflurane in oxygen at a flow rate of 0.8 l/min (5 % isoflurane for induction, 1.5–2.5 % isoflurane for maintenance) and kept on electronic heating pads to avoid hypothermia. Small-animal PET/CT imaging was performed with a dedicated small animal PET/CT scanner (Inveon®, Siemens Preclinical Solutions, Knoxville, TN). Two bed positions were acquired for CT and single bed position for PET to have an axial field of view (FOV, ∼10 cm) covering all major organs. The brain and neck were outside the FOV. A 18-min CT scan was carried out prior to the PET scan. Following the CT scan, a 90-min dynamic PET acquisition was started at the time of *i.v.* injection of [^11^C]preladenant (54 ± 20 MBq, *n* = 10). The emission sinograms were corrected for attenuation, scatter, and decay. The acquisition data was divided in 24 frames (6 × 10, 4 × 30, 2 × 60, 1 × 120, 1 × 180, 4 × 300, and 6 × 600 s). The data were reconstructed per time frame using an ordered set expectation maximization-3D/maximum a posteriori (OSEM3D/MAP) algorithm, with a voxel size of 0.39 × 0.39 × 0.80 mm^3^ and matrix of 256.

Immediately after the PET scan, animals were terminated by extirpation of the heart. Organs were harvested and measured with a well counter.

### Volumes of Interest

**Fig. 1:**
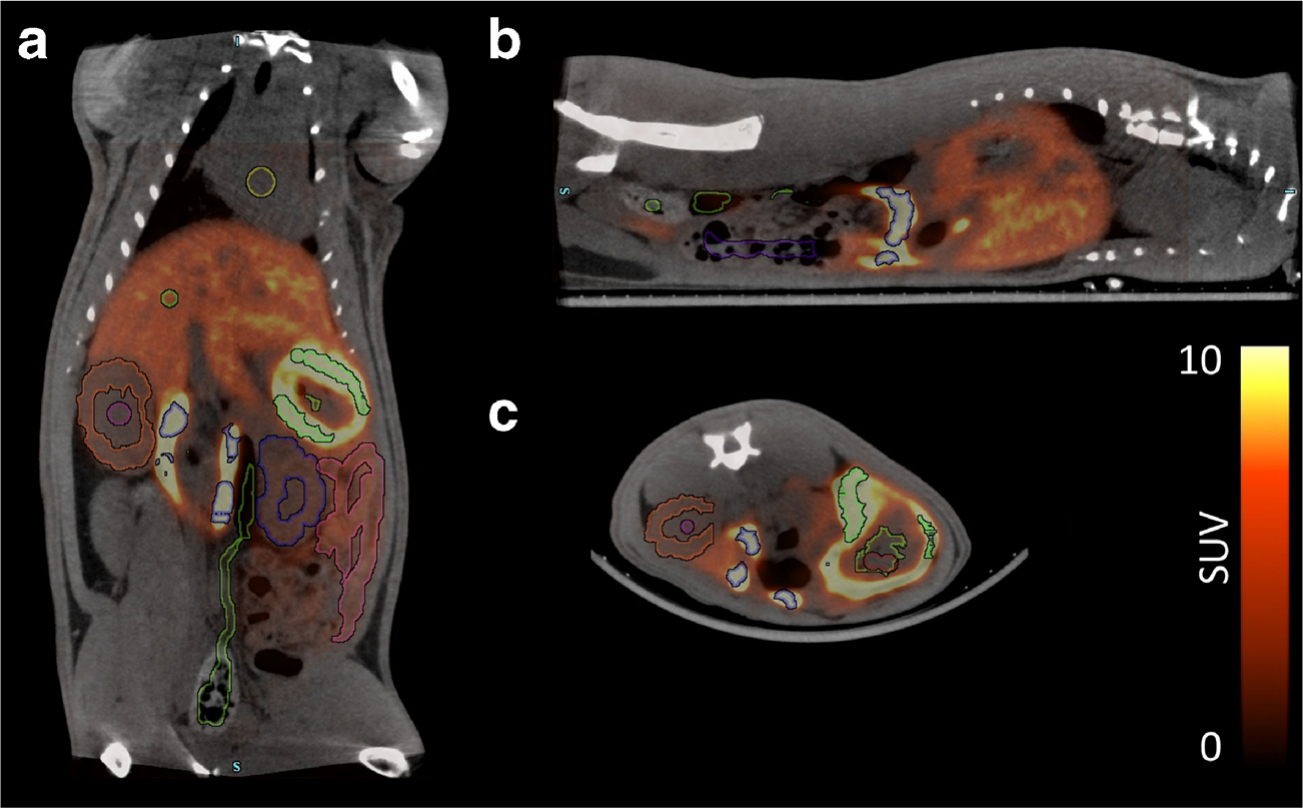
**a** Coronal, **b** saggital and **c** transversal view of a representative PET/CT scan with the manually defined volumes of interest.

In order to minimize the resolution-related PVE and spillover effects, spherical VOIs with diameters of 2.9–5.7 mm were placed at the center of the heart, lungs, liver, stomach content, spleen, pancreas, kidney contents, and testes on the PET/CT fusion images by a single observer, using Inveon Research Workplace (Siemens Medical Solutions, Knoxville, TN). For paired tissues such as the lungs, kidney contents, and testes, two VOIs were placed: one on the right and one on the left side. For the liver, VOIs were placed in two separate lobes. For the stomach wall, kidney wall, and intestines, VOIs were drawn well inside the boundary of the organs. The small intestine showed different tracer concentration at duodenum (first 8 % section of small intestine) and the rest of small intestine. Therefore, the two sections were delineated separately. Then, the total activity in the small intestine was calculated as total activity = 8 %*activity in duodenum + 92 %*activity in rest of small intestine. The urinary bladder was delineated based on the PET image. Examples of PET/CT co-registered images with manually defined VOIs are shown in Fig. 1 and Supplementary Fig. 2. The time-activity curves (TACs) were extracted from all VOIs, expressed as Becquerel per volume (Bq/ml) vs. time. Then, the Becquerel per volume was converted to Becquerel per mass (Bq/g), assuming organ density to be 1 g/ml for all organs except for lungs with a density of 0.33 g/ml [16].

### Organ Activity

The activity in the heart, lungs, liver, spleen, pancreas, and testes was calculated as activity concentration (Bq/g) multiplied by the mass of the organs (Table 1). The activity in kidney and stomach was the activity in organ walls plus the activity in organ contents. The masses of organ and organ contents were derived from a 300 g standard rat, based on the ex vivo biodistribution data of a group of 22 rats from a previous study (Hsd/Cpb:WU, Harlan, the Netherlands, 310 ± 20 g), by scaling their organ weights and the ratios of organ weight to body weight to that of an animal of 300 g (Table 1). The activity in intestines was obtained from the activity concentration (Bq/g)*mass of organ walls and contents, as the walls were indistinguishable from the contents. The activity in urinary bladder was computed as activity concentration (Bq/g)*mass of urinary bladder, where the mass was derived from the PET image (mass (g) = volume of bladder VOI (ml)*1 g/ml), with an average value of 0.29 g (Supplementary Fig. 2).

**Table 1:** Organ masses (mean ± SD) and organ-to-body mass ratios (O/B) of a 300-g standard male Wistar rat

Organ	Mass (g)	O/B (%)
Brain	1.95 ± 0.08	0.63
Heart	0.92 ± 0.09	0.31
Lung	1.25 ± 0.11	0.42
Liver	12.88 ± 0.92	4.29
Spleen	0.55 ± 0.05	0.18
Pancreas	0.98 ± 0.11	0.33
Kidneys	2.35 ± 0.13	0.76
Urinary bladder	0.07 ± 0.02	0.02
Testes	2.92 ± 0.19	0.97
Small intestine wall	1.95 ± 0.11	0.65
Colon wall	0.67 ± 0.23	0.22
Caecum wall	0.33 ± 0.06	0.11
Stomach wall	1.20 ± 0.21	0.40
Small intestine content	5.23 ± 2.00	1.74
Colon content	2.70 ± 1.20	0.90
Caecum content	3.17 ± 0.65	1.06
Stomach content	2.45 ± 1.36	0.82
*Kidney content	0.37 ± 0.03	0.12

*The mass was calculated (1 g = 1 ml) from the volume (0.374 ml) delineated on the PET/CT image (*n* = 3)

### Global Correction Factor (GCF)

The GCFs were generated to correct for PET imaging-derived activity in the lungs, urinary bladder and pancreas for spillover effects. The GCF was calculated by dividing the harvesting-based activity at 90 min post injection by the mean activity of the last frame (80–90 min) of PET imaging of the same animal. The PET imaging-derived TACs were corrected by multiplication with the GCF for all time points.

### Residence Time Calculation

The decay-corrected TACs were converted into TACs without decay correction, using the following formula: A = A_0_*2^[(t_0_-t)/T_1/2_], where A_0_ is the decay-corrected activity at time t, t_0_ is the time of injection. T_1/2_ is the half-life of ^11^C (= 0.34 h). The residence time (RT) in each organ was computed by integration of the area under the TAC (%ID vs. time). Area under the curve (AUC) between the start and the end of scan was calculated using trapezoidal numerical integration of AUC. The integral between the end of scan to the infinity was calculated as A_end_*0.49 h, where A_end_ is the activity at the end of scan, normalized to 1 MBq injected. The activity in tissues other than listed in Table 1 was grouped together as remainder. The remainder of the body was assumed to have a homogenous distribution of activity. The RT for the remainder was calculated by subtracting the RTs of all source organs from the theoretical total body RT of 0.49 h (= 0.34 h/ln2), assuming no excretion of activity.

The RTs for humans (RT_h_) were extrapolated from rat RTs (RT_r_) based on the difference of tissue-to-body mass ratio (Eq. 1), where O_h_ = human organ weight, O_r_ = rat organ weight, B_h_ = human body weight, B_r_ = rat body weight [17].

1 $$ R{T}_h=R{T}_r\times \left(\frac{O_h}{B_h}\right)/\left(\frac{O_r}{B_r}\right) $$

### Dosimetry Estimates

The dose calculations were in accordance with MIRD pamphlet No. 21 [18]. OLINDA/EXM 1.1 software [19] was applied to estimate organ-absorbed doses and the effective dose (ED) of a 70-kg reference adult phantom [20]. The doses were obtained without GCF correction. Equation 2 shows the absorbed dose (μSv/MBq) calculation of target organ i. The RT(j) is the RT in organ j (source organ), S (j,i) is the phantom-specific dose factor (S value) between the source organ j and target organ i. The EDs were computed based on tissue weighting factors from International Commission on Radiological Protection (ICRP) 60 [21] and ICRP 103 [22]. Ex vivo biodistribution data were calculated for average doses as well as maximum/minimum doses to assess worst/best case scenarios. The RTs used for obtaining maximum and minimum doses were computed from AUCs, where the maximum AUC was cumulative activity + 95 % confidence interval, and the minimum value was cumulative activity - 95 % confidence interval.

2 $$ \mathrm{Dose}(i)={\displaystyle \sum_jRT(j)\times S\left(j,i\right)} $$

## Results

**Fig. 2:**
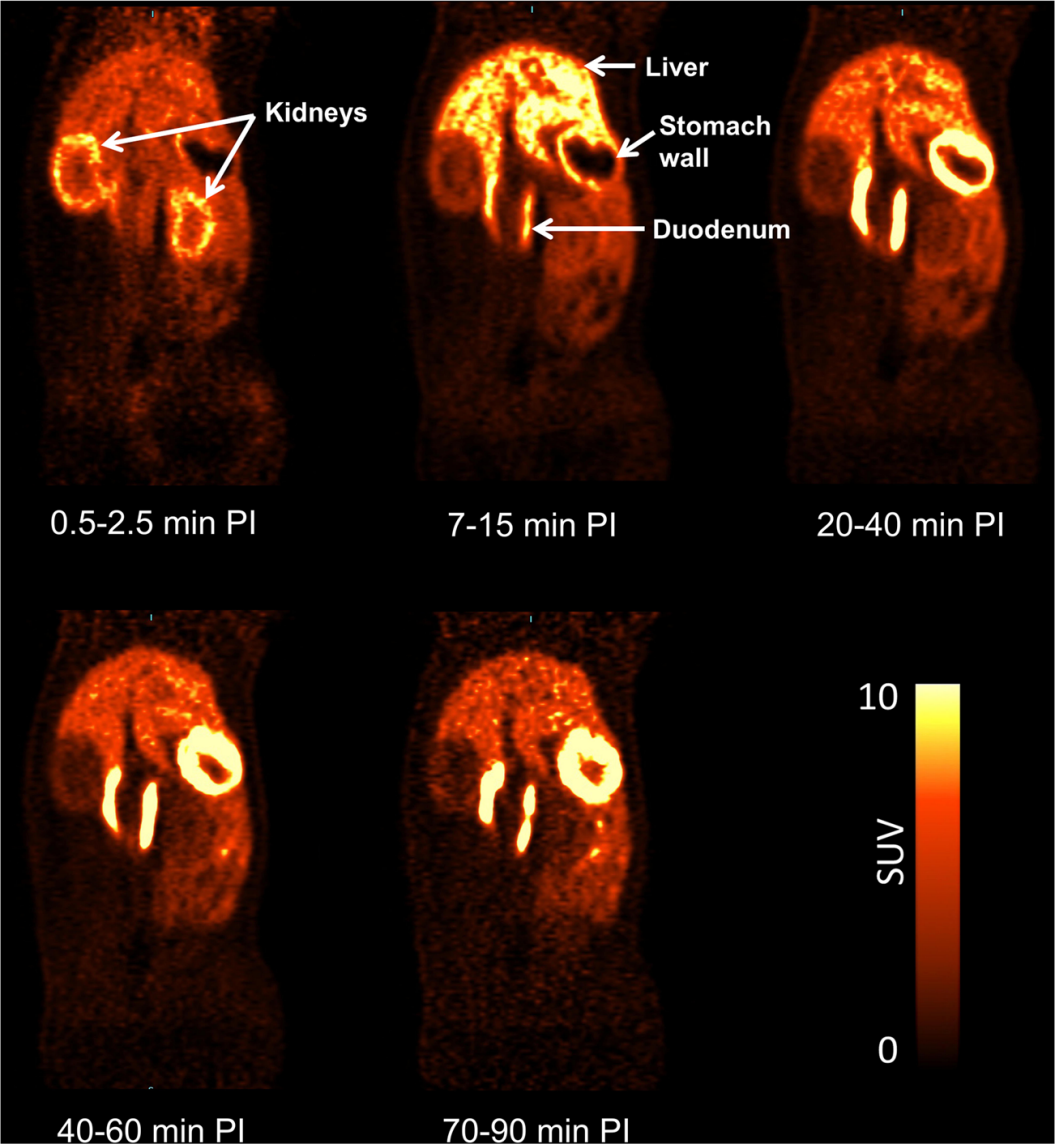
Representative coronal PET images of the distribution of [^11^C]preladenant in the rat body at different times post injection (PI).

**Fig. 3:**
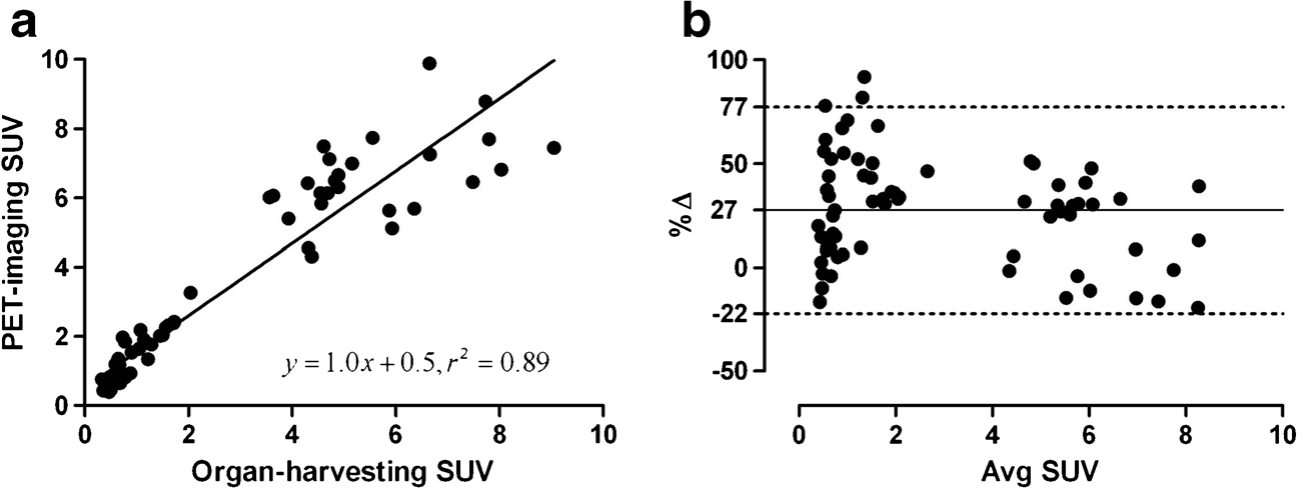
**a** Correlation between the tracer uptake in multiple organs (SUVs) derived from the last frame (80–90 min post injection) of PET-imaging and the tracer uptake obtained from organ harvesting of the same animals at 90 min post injection. **b** Bland-Altman analysis on the same set of data. The *solid line* shows an average bias of +27 %, the dashed lines represent the 95 % confidence intervals. Avg SUV = (SUV_Imaging_ + SUV_Harvesting_)/2, *Δ* = 2 × 100 × (*SUV*_Imaging_ − *SUV*_Harvesting_)/(*SUV*_Imaging_ + *SUV*_Harvesting_). (*n* = 10, data from the heart, liver, spleen, pancreas, kidney, testes, intestines, and stomach were used for comparison).

**Fig. 4:**
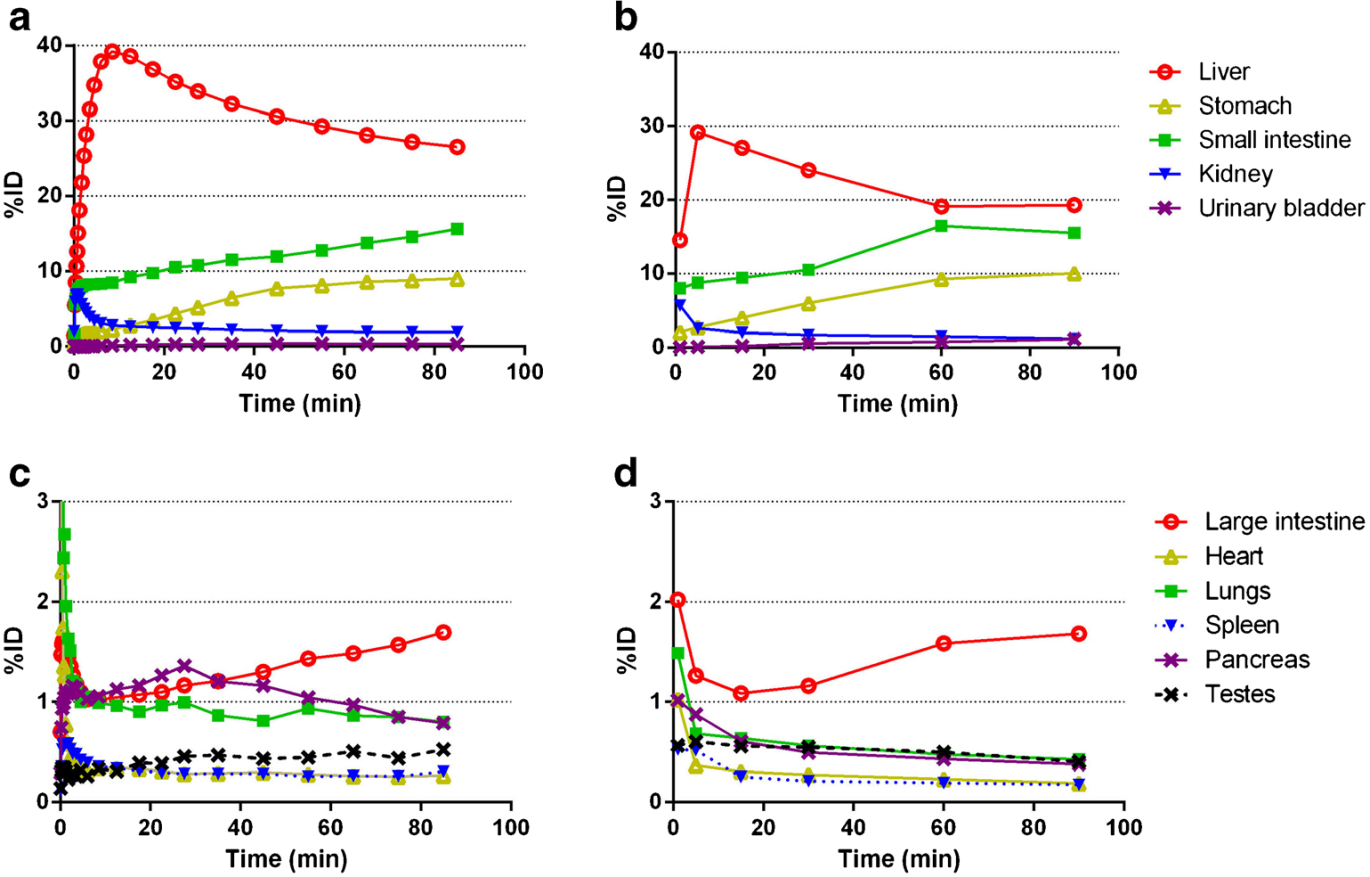
Decay-corrected mean time-activity curves for [^11^C]preladenant in multiple rat organs obtained from PET imaging (**a** and **c**) and organ harvesting (**b** and **d**).

Accumulation of [^11^C]preladenant in the stomach wall, liver, kidney, small intestine, and urinary bladder was clearly visual on the PET images (Figs. 1 and 2 and Supplementary Fig. 2). The highest tracer concentration was found in the duodenum and stomach wall, showing maximum SUVs of 28.4 ± 7.9 and 18.9 ± 3.1 (imaging data), respectively, at 90 min post injection. PET imaging and organ harvesting gave comparable SUVs, as is shown in Fig. 3. Figure 4 illustrates the dynamic distribution of [^11^C]preladenant in multiple rat organs derived from PET imaging and organ harvesting. The activity was corrected for decay to the time of injection. The highest total organ tracer uptake was found in the liver, with a peak of 39.2 ± 4.3 % injected activity at 8.5 min post injection for PET-imaging data, and 28.6 ± 9.5 % injected activity at 5 min post injection for ex vivo biodistribution data. The brain, kidneys, heart, lungs, spleen, and pancreas displayed rapid tracer clearance, with a peak uptake within 1 min. The small intestine, large intestine, stomach, and urinary bladder exhibited continuously increasing uptake for the total scan duration of 90 min.

The residence times and the percentage of injected dose for the human adult model derived from rat organ activity are listed in Table 2. The highest cumulative activity was found in the liver and small intestine, with RTs of 9.43E-02 ± 0.63E-02 h and 7.03E-02 ± 1.95E-02 h for imaging-based data, and 7.26E-02 h and 7.64E-02 h for organ harvesting-based liver and small intestine data, respectively. The data indicated that the tracer was mainly excreted by the hepatobiliary system, as this accounted for the excretion of >30 % of activity, whereas merely <1.2 % activity was found in the urinary bladder (no voiding). The imaging-based RTs were in agreement with organ harvesting-based values in general, with a mean difference of 24 % (range 6–27 %, except for the lungs, pancreas, and urinary bladder, with differences of 48, 53, and 60 %, respectively). When the GCFs were applied to imaging-derived TACs of the lungs, pancreas, and urinary bladder, the resulting RT values for the lungs and pancreas became comparable with organ-harvesting-derived RTs, with differences below 17 %. However, this method was not valid for urinary bladder, as the difference between methods increased to 80 % after correction (Supplementary table 1).

**Table 2:** Human residence times (RTs, in Becquerel-hour per Becquerel injected) and percent inject dose (%ID) estimates for [^11^C]preladenant based on PET-imaging and average organ-harvesting RTs from rats

	RT_Imaging (mean ± SD)	%ID_Imaging	RT_Harvesting	%ID_Harvesting
Testes	1.13 × 10^−04^ ± 0.30 × 10^−04^	0.02	1.49 × 10^−04^	0.03
Pancreas	2.09 × 10^−03^ ± 0.50 × 10^−03^	0.43	1.22 × 10^−03^	0.25
Spleen	2.25 × 10^−03^ ± 0.56 × 10^−03^	0.46	2.07 × 10^−03^	0.42
Heart	2.58 × 10^−03^ ± 0.45 × 10^−03^	0.53	2.43 × 10^−03^	0.49
Urinary bladder	3.15 × 10^−03^ ± 1.78 × 10^−03^	0.64	5.88 × 10^−03^	1.20
Lower large intestine wall	3.43 × 10^−03^ ± 0.99 × 10^−03^	0.70	4.31 × 10^−03^	0.88
Upper large intestine wall	4.52 × 10^−03^ ± 1.30 × 10^−03^	0.92	5.67 × 10^−03^	1.16
Kidneys	7.15 × 10^−03^ ± 0.89 × 10^−03^	1.28	5.94 × 10^−03^	1.21
Stomach	1.18 × 10^−02^ ± 0.19 × 10^−02^	2.40	1.41 × 10^−02^	2.87
Lungs	1.76 × 10^−02^ ± 0.28 × 10^−02^	3.58	1.07 × 10^−02^	2.19
Small intestine	7.03 × 10^−02^ ± 1.95 × 10^−02^	14.34	7.64 × 10^−02^	15.58
Liver	9.43 × 10^−02^ ± 0.63 × 10^−02^	19.23	7.26 × 10^−02^	14.79
Brain	NA	NA	6.84 × 10^−03^	1.39
Remainder	2.72 × 10^−01^ ± 0.25 × 10^−01^	55.48	2.82 × 10^−01^	57.53

The highest organ dose was received by the small intestine (imaging: 23.2 μSv/MBq, harvesting: 25.1 μSv/MBq), and followed by the liver (imaging: 16.8 μSv/MBq, harvesting: 15.1 μSv/MBq), stomach wall (imaging: 8.9 μSv/MBq, harvesting: 12.5 μSv/MBq), and the kidneys (imaging: 8.4 μSv/MBq, harvesting: 7.2 μSv/MBq) (Table 3). ED estimates were similar between PET-imaging and organ-harvesting methods, being 5.1 ± 0.3 μSv/MBq for PET-imaging, and 5.1 μSv/MBq for organ-harvesting, with a range between 4.4 and 5.8 μSv/MBq (ICRP 103). When the weighting factors from ICRP 60 were used in the calculation, the resulting EDs were 5.5 ± 0.4 μSv/MBq for PET-imaging, and 5.6 μSv/MBq for organ-harvesting. When the source organs listed in Table 2 were reduced to organs with uptake visually higher than the background on the PET image (stomach, liver, kidney, small intestine, urinary bladder, lungs (lungs were not visible on the PET image, however the activity concentration in lungs was 3.3 times higher than presented on the image. Therefore, lung was also included as a source organ); Figs. 1 and 2), the EDs based on RT values of lungs and visible source organs only were 4.7 ± 0.2 μSv/MBq for PET-imaging, and 4.6 μSv/MBq for organ-harvesting, based on ICRP 103, and was 5.1 μSv/MBq for both PET-imaging and organ-harvesting, based on ICRP 60.

**Table 3:** Human organ-absorbed doses (μSv/MBq) and effective doses (μSv/MBq)

Organ	PET-imaging	%COV	Harvesting_AVG	Harvesting_Max	Harvesting_Min
Adrenals	3.4	2.6	3.1	3.1	3.2
Brain	1.5	9.1	1.9	2.0	1.7
Breasts	1.8	5.8	1.8	1.6	2.0
Gallbladder wall	4.9	3.2	4.6	4.8	4.2
Lower large intestine wall	6.1	13.6	7.0	7.9	6.1
Small intestine	23.2	24.4	25.1	31.2	18.1
Stomach wall	8.9	10.7	10.1	12.5	7.5
Upper large intestine wall	7.4	12.0	8.3	9.5	6.9
Heart wall	3.8	9.7	3.5	3.7	3.2
Kidneys	8.4	10.0	7.2	7.9	6.3
Liver	16.8	6.3	13.2	15.1	10.7
Lungs	5.7	11.7	3.9	4.2	3.5
Muscle	2.2	3.6	2.2	2.1	2.4
Ovaries	3.9	7.8	4.2	4.5	3.9
Pancreas	8.2	16.2	5.8	6.5	5.0
Red marrow	2.2	1.3	2.3	2.2	2.3
Osteogenic cells	2.8	6.2	2.9	2.6	3.3
Skin	1.6	6.1	1.6	1.5	1.8
Spleen	4.9	16.4	4.6	6.0	3.2
Testes	1.5	10.1	1.9	1.9	1.8
Thymus	2.0	6.3	2.0	1.8	2.3
Thyroid	1.8	8.3	1.9	1.6	2.2
Urinary bladder wall	4.4	27.2	6.3	7.6	4.9
Uterus	3.6	7.0	3.9	4.1	3.7
Total body	2.9	0.2	2.8	2.8	2.9
Effective dose (ICRP 60)	5.5	6.8	5.6	6.3	4.7
Effective dose (ICRP 103)	5.1	6.0	5.1	5.8	4.4

*COV* coefficient of variation, calculated as SD/mean, *AVG* average, *Max* maximum, *Min* minimum

## Discussion

Preclinical radiation dosimetry is a common approach to estimate the activity that would be absorbed by humans in a clinical study. Our study has shown that the ED of [^11^C]preladenant (5.5 μSv/MBq, ICRP 60) projected from the rat biodistribution data is within the same range as other C-11 labeled PET tracers (3.0–16.0 μSv/MBq, mean 5.1 μSv/MBq) [23]. The dose limiting organ for [^11^C]preladenant administration is the small intestine. A PET study with [^11^C]preladenant can be performed in human subjects using a maximum injection dose of 2.0 GBq based on the dose limit (50 mGy) to the small intestine (Radioactive Drug Research Committee criteria). When the maximum ED of 10 mSv is taken into account [24], the maximum administered dose will be 1.8 GBq.

The conventional organ-harvesting method for radiation dosimetry estimates has disadvantages such as laborious sampling procedure, limited information obtained from single animals, and low time resolution of the dynamic data, resulting in experimental measured organ doses that deviate from the true values. Small-animal PET imaging could overcome these problems and serve as an alternative to organ harvesting to study in vivo tracer biodistribution, provided PVE and spillover effects are minimal. Our study used a high-resolution small-animal PET camera with a spatial resolution of 1.35 mm at the center of the FOV. Furthermore, PET imaging was performed on rats, which have larger organ sizes than mice. Therefore, it is possible to define VOIs containing a sufficient number of voxels well inside the boundary of organs to ensure sufficient counting statistics. In addition, high-resolution CT images were co-registered with PET images of the same animal that could serve as anatomical references for organ delineation (Fig. 1). Taken together, these approaches could substantially reduce PVE/spillover effects due to the limited spatial resolution of the PET camera. Thus, the reliability of PET-imaging data can be greatly improved. Indeed, when we compared the PET-imaging-derived activity uptake with activity uptake obtained from organ-harvesting, a high degree of concordance was found between the two measures (Fig. 3a). A good correlation was observed by a linear regression model, with a slope equal to 1.0, and a correlation (*r* ^2^) of 0.89 (*n* = 10).

Our PET-imaging results, albeit superior to other published data in terms of high agreement with organ-harvesting measurements [12–14], still showed a small deviation from organ-harvesting data. As is shown in Fig. 3b, a positive bias was observed for PET-imaging, especially in regions with low uptake. We assume that the overestimation of activity in several organs with low uptake, such as the spleen, pancreas, lungs, and kidneys, is due to spillover effects, as these organs are adjacent to regions with high uptake, like the stomach wall, liver and duodenum. Therefore, one can easily imagine that the pancreas would be the most affected organ, since it has a small volume, and is situated between organs with the highest activity. Activity in the lungs measured by PET-imaging also deviated substantially from activity uptake obtained from organ-harvesting. Spillover of activity from the liver would be one explanation. This spillover effect was further enhanced by the 0.33 g/ml conversion factor that was used to calculate the activity concentration in the lungs. Thus, the spillover effect was tripled during the calculation. Organs with high uptake seem to be less affected by PVE, as the PET-imaging and organ-harvesting estimated comparable values. Interestingly, we found that the activity in the liver was higher measured with PET-imaging than organ-harvesting. It is unlikely that the activity measurement with PET was affected by spillover effect, as the VOIs in the liver were far away from surrounding tissues (Fig. 1). Here we consider that the activity in the liver is more reliable with PET-imaging than organ-harvesting, because the body fluid loss in the liver during the ex vivo biodistribution process might lead to an underestimation of activity with organ-harvesting. As discussed above, the activity was most concentrated in the stomach wall and duodenum; therefore, the hepatic portal vein (75 % of liver blood supply) and intrahepatic fluid may contain high levels of activity, maybe even higher than the activity in hepatocytes. When the heart was extirpated, the blood, which makes up 30 % of total volume in the liver, is drained way. The intrahepatic activity may also be taken away along with blood loss. Therefore, the activity measured with ex vivo biodistribution could be underestimated.

Despite the overestimation of activity with PET-imaging in several organs with low activity uptake, an average bias of +27 % is quite acceptable, because organs with low activity uptake do not contribute much to the radiation dose estimation. The bias is much smaller in organs with high activity uptake, which are more important source organs for dose estimation (Fig. 3). Furthermore, the cumulative activity (AUC) estimates by both methods were similar, with the exceptions of the pancreas, lungs, and urinary bladder, as is shown in Table 2. The results suggest that the spillover effect is the major factor leading to an overestimation of activity with PET-imaging. However, such deviations with PET-imaging were small and have been greatly reduced by the strategies discussed above, in particular by drawing small VOIs with regular shapes well inside the boundary of organs and by dissecting hollow organs with contents.

The activity in urinary bladder was substantially underestimated by 47 % with PET-imaging (Table 2). The organ mass of the urinary bladder (0.29 g on average) calculated from the volume of this organ on the PET images was different from either the mass of urinary wall (0.07 g) or the urinary wall with urine (0.5–4.5 g) measured with organ-harvesting. The volume of the urinary bladder cannot be properly estimated by PET because of the low activity in urine. The activity might have a higher concentration around the ureter, where the urine was delivered from the kidneys, making the small area visible from the background. However, a large portion of urinary bladder and urine was not visible, resulting in an underestimation of activity as only the visible portion was considered in the calculation. However, since the activity was low in the urinary bladder, the inaccurate measurement with PET-imaging would not significantly impair the dose estimation, as the urinary bladder was neither a source organ, nor a critical organ in this case.

In order to reduce PET resolution-associated PVE and spillover effect, several methodologies have been applied to improve data accuracy, such as region-based PVE correction reported by Lehnert, et al. [25], and the use of GCFs based on ex vivo biodistribution data, as was suggested by Kesner, et al. [13]. The former technique is robust and resulted in good recovery of activity in small VOIs. However, it requires accurate anatomic information. Since not all structures are identifiable with CT imaging, and the shapes of the organs can be different between scans, it becomes very complicated or even impossible to apply this methodology to the whole-body scan. A simpler method is the GCF correction which was tested in our study. The major limitation of this method is that it does not change the shape of the TACs in organs after correction, whereas the PVE/spillover effect has a dynamic impact on TACs, as it is affected by the activity kinetics in adjacent tissues. The global correction failed to correct the activity in urinary bladder, since the mismatch of activity in this organ with PET-imaging was not due to PVE. Since the uptake in pancreas and lungs was affected by spillover from the liver, GCF correction substantially improved the results in these organs. Other organs showed comparable results between PET-imaging and organ-harvesting, indicating that the PVE was small. Considering the disadvantages of global correction, which may introduce a bias and impair the PET-imaging results, and considering the reliability of organ-harvesting data, especially in the liver, global correction was not used in the dose calculations. Even without global correction the ED and critical organ dose estimates were similar between PET-imaging and organ-harvesting, indicating that the PVE correction is not crucial in our dosimetry study. Therefore, ex vivo biodistribution after PET scanning can be omitted.

In our study, the head and neck regions were not scanned because (1) the head and neck were not expected to show relevantly high uptake (based on ex vivo biodistribution and our previous studies); thus, tracer uptake in these regions had very small contribution to the dose calculation. (2) Technical hurdles. The PET/CT modality requires manual positioning of animals on both PET and CT sides when whole-body PET scan is performed, resulting in difficulty in coregistration of PET and CT images. Furthermore, PET scanning with continuous bed motion leads to dynamic images with low time-resolution and low statistical quality. In our particular case, the impact on dosimetry of including the head and neck region was deemed too small to justify its cumbersomeness and reduced image quality. In case of the brain and neck as possibly critical organs, PET imaging of these regions is necessary and is best possible with our PET/CT system by scanning each animal twice with one scan on the head + upper torso and the other on the lower part of the body.

## Conclusions

The ED of [^11^C]preladenant is comparable with other C-11 labeled PET tracers. A single injection dose of 370 MBq (10 mCi) should be easily allowed for the first PET study in humans. Estimation of the radiation dosimetry of [^11^C]preladenant by PET/CT imaging in rats is feasible and gives comparable results as organ harvesting, provided that small VOIs with regular shapes are used and the content of hollow organs is taken into account. Dosimetry by PET-imaging can thus strongly reduce the number of laboratory animals required.

## Electronic supplementary material

Below is the link to the electronic supplementary material.

ESM 1 (PDF 121 kb)
