# Supplementary material for: Radiation Dosimetry of a Novel Adenosine A2A Receptor Radioligand [11C]Preladenant Based on PET/CT Imaging and Ex Vivo Biodistribution in Rats
Source: Mol Imaging Biol. 2016 Aug 18;19(2):289–97. doi: 10.1007/s11307-016-0992-3 (PMC5336543; doi:10.1007/s11307-016-0992-3)
Supplement: Supplementary file 1 — (PDF 121 kb) [file 11307_2016_992_MOESM1_ESM.pdf]

Electronic Supplementary Material

Radiation Dosimetry of a Novel Adenosine A_{2A} Receptor Radioligand [^{11}C]Preladenant Based on PET/CT Imaging and *Ex Vivo* Biodistribution in Rats

Journal: Molecular Imaging and Biology

Xiaoyun Zhou, Philip H. Elsinga, Shivashankar Khanapur, Rudi A.J.O. Dierckx, Erik F.J. de Vries and Johan R. de Jong

Department of Nuclear Medicine and Molecular Imaging, University of Groningen, University Medical Center Groningen, Groningen, The Netherlands.

Running foot line: preclinical dosimetry for [^{11}C]preladenant

Manuscript category: Article

Corresponding author: Johan R. de Jong, E-mail j.r.de.jong@umcg.nl, Telephone +31-50-3615875, Telefax +31-50-3611687

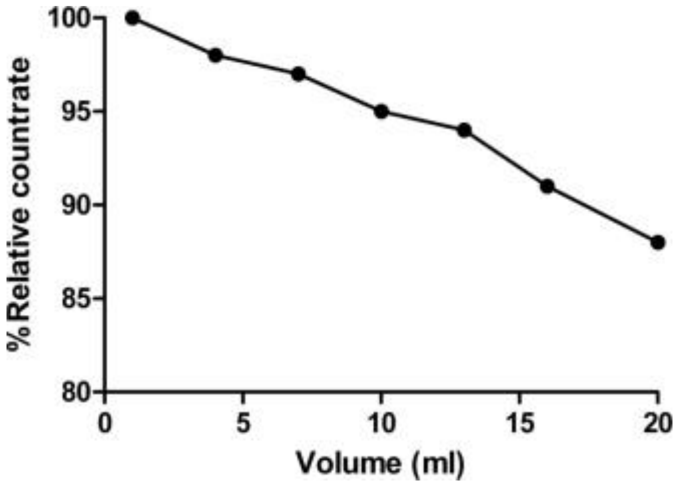

Supplementary Figure 1 %Relative countrate against sample volume measured with the PerkinElmer well counter.

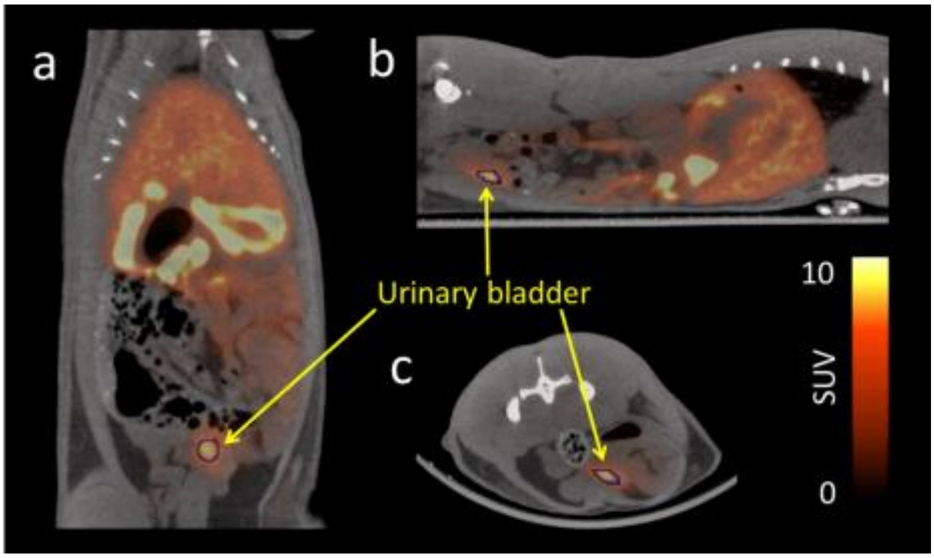

Supplementary Figure 2 Coronal (a), saggital (b) and transversal (c) view of urinary bladder VOI on a representative PET/CT image.

Supplementary table 1 Comparison of residence times (in Becquerel-hour per Becquerel injected) of [^{11}C]preladenant in pancreas, urinary bladder and lungs between PET imaging, PET imaging with global factor correction, and *ex vivo* biodistribution.

	Imaging (mean±SD)	Imaging with correction (mean±SD)	Harvesting
Pancreas	2.09E-03±0.50E-03	1.09E-03±0.4.8E-03	1.22E-03
Urinary bladder	3.15E-03±1.78E-03	1.39E-02±0.90E-02	5.88E-03
Lungs	1.76E-02±0.28E-02	9.04E-03±1.65E-03	1.07E-02
